# Caregiver contribution to patient self‑care and quality of life among informal carers of adult patients with inflammatory bowel disease: a cross‑sectional study

**DOI:** 10.1007/s11136-026-04305-w

**Published:** 2026-06-15

**Authors:** Daniele Napolitano, Maria Francisca Murgiano Gonzalo, Alessio Lo Cascio, Fabrizio Benedetti, Arianna Povoli, Teresa Sanità, Valeria Suriano, Greta Lorenzon, Arianna Luongo, Debora Zaetta, Francesca De Marinis, Nicoletta Orgiana, Giulia Petruccini, Valentina Vanzi, Francesco Pastore, Adriana Rivera-Sequeiros, Mattia Bozzetti

**Affiliations:** 1 SITRA and Scientific Direction- Fondazione Policlinico Universitario A. Gemelli IRCCS, Largo Agostino Gemelli 8, 00168 Rome, Italy; 2 Department of Gastroenterology, Hospital del Mar, Barcelona, Spain; 3 Direction of Health Professions, La Maddalena Cancer Center, 90146 Palermo, Italy; 4 Department of Biomedicine and Prevention, Tor Vergata University, 00133 Rome, Italy; 5 Department of Gastroenterology, Azienda Sanitaria Universitaria Friuli Centrale, 33100 Udine, Italy; 6 A.O.U. Az.Ospedaliera Consorziale Policlinico Bari, U.O. Gastroenterologia Universitaria, Bari, Italy; 7 Department of Surgery, Oncology and Gastroenterology (DISCOG), University Hospital of Padua, Padua, Italy; 8 Department of Gastroenterology, Ospedale del Mare, ASL NA1 Centro, 80147 Naples, Italy; 9 Santa Maria del Prato Hospital, Feltre, Italy; 10 Gastroenterology and Digestive Endoscopy, Nuovo Regina Margherita Hospital, Rome, Italy; 11 CUT - Fondazione Policlinico Universitario “A. Gemelli” IRCCS, Rome, Italy; 12 Center of Excellence for Nursing Scholarship (CECRI), Board of Nursing (OPI) of Rome, 00136 Rome, Italy; 13 Nephrology, Dialysis and Transplantation Unit, Department of Precision and Regenerative Medicine and Jonian Area - (DiMePRe-J), University of Bari Aldo Moro, Bari, Italy; 14 Research Unit, Virgen Macarena University Hospital, 41009 Seville, Spain; 15 Direction of Health Professions, ASST Cremona, 26100 Cremona, Italy

**Keywords:** Inflammatory bowel disease, Informal caregivers, Self-care, Quality of life, Generalized additive models, Mental health

## Abstract

**Purpose:**

Informal caregivers play a critical role in the management of adult patients with inflammatory bowel disease (IBD), yet the association between their contribution to patient self-care and their own health-related quality of life (HRQoL) remains underexplored. This study aimed to examine the cross-sectional association between caregivers’ contribution to patient self-care and caregivers’ physical and mental HRQoL.

**Methods:**

A multicentre cross-sectional study was conducted across nine IBD centres in Italy. Caregiver contribution to patient self-care was assessed using the Caregiver Contribution to Self-Care of Chronic Illness Inventory (CC-SC-CII), including Maintenance, Monitoring, and Management domains. HRQoL was measured using the 12-Item Short Form Survey, yielding Physical Component Summary (PCS-12) and Mental Component Summary (MCS-12) scores. Generalized Additive Models were used to estimate potential non-linear associations between CC-SC-CII domains and HRQoL.

**Results:**

The study included 275 informal caregivers. No statistically supported associations were observed between CC-SC-CII Maintenance, Monitoring, or Management and PCS-12. For MCS-12, only CC-SC-CII Maintenance showed a statistically supported non-linear association (edf = 4.34, F = 2.21, *p* = 0.018; adjusted R^2^ = 0.272; deviance explained = 38.1%). The model-estimated MCS-12 difference between the 75th and 25th percentiles of CC-SC-CII Maintenance was 6.58 points (95% CI 1.50–11.70). No statistically supported associations were observed for CC-SC-CII Monitoring or Management and MCS-12.

**Conclusion:**

Caregiver contribution to patient self-care maintenance was associated with caregivers’ mental, but not physical, HRQoL. The findings should be interpreted as exploratory associations and require confirmation in longitudinal dyadic studies including both caregiver- and patient-reported outcomes.

**Supplementary Information:**

The online version contains supplementary material available at https://doi.org/10.1007/s11136-026-04305-w.

## Introduction and background

Inflammatory bowel disease (IBD), encompassing Crohn’s disease (CD) and ulcerative colitis (UC) is a chronic immune-mediated disorder characterised by alternating periods of remission and exacerbation. Over the past three decades, both incidence and prevalence have increased worldwide; Modeling studies predict that up to 1% of the population in highly industrialised countries will live with IBD by 2030 [5, 6]. The global burden of inflammatory bowel disease (IBD) continues to rise, particularly in high-income countries. Population based forecasting studies predict that by 2030, IBD prevalence may approach 1% of the population in some regions. For instance, Canadian projections estimate a prevalence of 981 cases per 100,000 inhabitants (0.98%), whereas data from the EPIMAD cohort in Northern France suggest that approximately 0.6% of the population will be affected by IBD by 2030. These findings underscore the growing public health impact of IBD and the increasing demand for long-term disease management strategies. [7, 30]. Although pharmacological advances have improved remission rates, IBD remains incurable, so long-term management aims to minimise symptoms, prevent complications, and sustain health related quality of life (HRQoL).

Self-care, the set of actions undertaken to maintain health, monitor symptoms, and manage disease-related changes, has become a cornerstone of managing chronic illnesses. The Middle-Range Theory of Self-Care of Chronic Illness organises self-care into three components: maintenance (behaviours that sustain physical and emotional stability), monitoring (ongoing surveillance for symptoms or changes) and management (responsive actions when changes occur), and highlights factors such as experience, motivation, cognitive function and social support that influence performance [28]. In IBD, self-care activities include adhering to complex medication regimens, making dietary adjustments, tracking symptoms, and seeking timely medical advice. Evidence from patient-focused studies suggests that better self-care is associated with fewer flare-ups and improved HRQoL; however, research in IBD remains limited, and few studies have examined how patients and caregivers enact and support these behaviors [21, 22, 24].

Self-care is rarely performed in isolation. During exacerbations of IBD, family members and friends often assume informal caregiving roles: they administer medications, manage nutrition, coordinate appointments, and provide emotional support. Within the framework of the Middle-Range Theory, these activities constitute a “caregiver contribution to patient self-care,” whereby caregivers assist with maintenance (for instance, preparing appropriate meals and reminding patients to take medicines), monitoring (noticing changes in symptoms), and management (helping decide when to contact healthcare providers). Although such dyadic interactions may influence disease outcomes, caregivers themselves are vulnerable to significant burden. Cross-sectional evidence indicates that almost half of caregivers of adults with IBD report high burden; this burden is associated with younger age, female sex, lower income, and more severe disease in the patient, whereas participation in religious or support activities appears protective [26]. An integrative review concluded that the chronic and relapsing nature of IBD exposes family caregivers to biopsychosocial, physical, and financial strain and called for family-centred interventions [20]. Other observational studies indicate that approximately 40% of caregivers experience a significant burden, and those with high burden miss more work and exhibit reduced productivity. Predictors of this burden include patient disease severity and the hours spent caregiving [47]. Psychological resources also matter: resilience has been shown to mediate the relationship between caregiver burden and hope among caregivers of IBD patients [49]. These findings underscore caregiver vulnerability but do not clarify how their own self-care behaviours, or the extent of their contribution to patient self-care, affect their HRQoL.

Insights from other chronic diseases illustrate the importance of examining caregiver contributions to patient self-care. In heart failure, longitudinal mediation analyses reveal that caregiver contributions to patient self-care directly improve patient self-care and, through patient self-care, indirectly enhance patient HRQoL, whereas interventions such as motivational interviewing increase caregiver preparedness and reduce patient symptom burden [4, 16, 17]. In oncology, conceptual and empirical work indicates that caregiver well-being directly and indirectly influences care quality through its effects on caregiver contributions and patient self-care management [15]. Studies involving caregivers of patients with advanced cancer demonstrate that psychological distress mediates the relationship between caregiver burden and HRQoL and that family resilience moderates this pathway; caregivers with high burden engage less in self-care and display lower resilience [8, 38]. Collectively, these findings suggest that caregiver contributions to patient self-care are recognised as determinants of well-being and clinical outcomes in other diseases, and that targeted interventions have been developed to support caregivers.

Despite these advances, research on IBD is still sparse. Only a handful of studies have explored caregivers’ experiences, and none have systematically evaluated how caregivers’ contribution to patients’ self-care or their contributions to patient self-care influence their HRQoL. Existing protocols, such as IBD-SELF, focus on patient self-care and measure caregiver contributions but do not treat caregivers’ contribution to self-care as a distinct construct [24]. Moreover, recent systematic reviews of self-care management interventions in IBD indicate that available programmes are largely patient-focused, with few non-medical interventions and uncertain effects on behaviour and outcomes [11]. Research on resilience in IBD patients emphasises its critical role in effective self-care management and suggests that enhancing resilience may benefit both patients and caregivers [3, 19]. However, whether caregivers’ own self-care practices, self-efficacy, or resilience independently influence their HRQoL, and how these factors interact with the support they provide to patients, remains unexplored.

Evidence consistently shows that this contribution acts as a proximal determinant of caregivers’ HRQoL, directly and through reduced psychological burden [25]. Psychosocial stress models further suggest that contributing enhances perceived control and meaning, buffering distress, whereas unmet demands heighten strain and exhaustion, ultimately compromising well-being [18].

Guided by dyadic self-care frameworks, we considered caregiver contribution, patient disease activity, care load, and caregivers’ HRQoL as interrelated components of the caregiving context. In chronic illness, caregiver contribution may support patient self-care and adherence, but disease activity and care demands may also shape the intensity and meaning of caregiving involvement [36 ]ref [4, 9, 12]. Therefore, in the present cross-sectional study, disease activity and care load were interpreted as contextual variables measured at enrolment rather than as confirmed mediators or temporally ordered consequences of caregiver contribution. Directed Acyclic Graphs (DAGs) were used to make these assumptions explicit and to guide covariate selection for the primary association between caregiver contribution and caregivers’ HRQoL, with the aim of reducing the risk of inappropriate adjustment, overadjustment, or collider bias [33, 34].

This study was guided by the following research question: among informal caregivers of patients with IBD, how is caregiver contribution to patient self-care associated with caregivers’ physical and mental HRQoL at a single time point?

The study had two objectives. First, we aimed to describe caregiver contribution to patient self-care, measured across the Maintenance, Monitoring, and Management domains, and caregivers’ physical and mental HRQoL. Second, we aimed to estimate the cross-sectional associations between each caregiver contribution domain and caregivers’ PCS-12 and MCS-12 scores, allowing for potential non-linear relationships.

## Methods

### Study design and methodological framework

A multicentre cross-sectional study was conducted between April and June 2024 across nine Italian IBD centres. The methodological framework was adapted from the IBD-SELF protocol [24], a longitudinal observational protocol designed to investigate self-care in patients with IBD and caregiver contribution to patient self-care. The present study differs from the original longitudinal design because it focuses specifically on informal caregivers and uses data collected at a single baseline time point.

### Reporting and transparency

This study is reported in accordance with the Strengthening the Reporting of Observational Studies in Epidemiology (STROBE) guidelines for cross-sectional studies. A completed STROBE checklist is provided as Supplementary Material [41].

### Participants and setting

Caregivers of patients were recruited from gastroenterology outpatient clinics participating in the IBD-SELF study across nine IBD Units in Italy. Eligible caregivers were adults (≥ 18 years) identified by a patient with CD or UC as their principal unpaid caregiver. Inclusion required sufficient proficiency in Italian to complete questionnaires and to provide written informed consent. Paid professional caregivers and individuals with cognitive impairments that preclude their ability to complete questionnaires were excluded. Convenience sampling was used during routine patient appointments to enroll participants.

### Variables and measures

#### Caregiver contribution to patient self-care

Caregiver contribution to patient self-care was assessed using the Caregiver Contribution to Self-Care of Chronic Illness Inventory (CC-SC-CII) [39, 40] Supplementary File 1. The CC-SC-CII measures how frequently caregivers contribute to patient self-care behaviours across three domains: Maintenance, Monitoring, and Management. Maintenance refers to routine behaviours aimed at maintaining patient stability, such as supporting adherence to treatment and health-promoting routines. Monitoring refers to the caregiver’s contribution to observing symptoms and detecting changes in the patient’s condition. Management refers to the caregiver’s contribution to decision-making and actions undertaken in response to symptoms or clinical changes.

Items are rated on a five-point Likert scale ranging from 1 = “never” to 5 = “always.” Domain scores are standardised to a 0–100 scale, with higher scores indicating more frequent caregiver contribution to patient self-care (a score of 70 or above is commonly interpreted as adequate caregiver contribution to patient self-care). Reliability estimates were satisfactory across the three domains, with omega values ranging from 0.82 to 0.96 [39].

#### Quality of life

Caregivers’ HRQoL was assessed using the validated Italian version of the 12-Item Short Form Survey [42] [14]. The SF-12 yields two summary scores: the Physical Component Summary (PCS-12), reflecting perceived physical health, and the Mental Component Summary (MCS-12), reflecting perceived mental health. Both scores are standardised to a mean of 50 and a standard deviation of 10 in the reference population, with higher scores indicating better perceived health status.

#### Sociodemographics

Sociodemographic and caregiving-context information was collected via self-report to describe the caregiver sample and to inform the DAG-based covariate-selection framework. Sociodemographic variables included caregiver age, sex, education level, and employment status. Age was treated as a continuous variable, sex was recorded as female or male, education level was classified as primary school, middle school, high school, or bachelor’s degree. Employment status was classified as worker or non-worker, including homemakers, retired persons, and other non-employed categories.

Caregiving-context variables included relationship to the patient, time as caregiver, hours of caregiving per week, care load, and patient disease activity at enrolment. Relationship to the patient was classified as partner/spouse, parent/sibling, other family member, or friend/other. Time as caregiver was classified as less than 1 year, 1–2.9 years, 3–4.9 years, or 5 years and over. Hours of caregiving per week were collected as a caregiver-reported estimate of the time usually dedicated to caregiving activities.

Care load was assessed as a contextual indicator of caregiving intensity and classified as mild, moderate, or severe based on the reported time dedicated to caregiving activities and the level of involvement in disease-related support. Mild care load referred to limited caregiving involvement with occasional support activities and lower time commitment. Moderate care load referred to regular caregiving involvement with recurrent participation in treatment routines, appointments, or symptom-related support. Severe care load refers to intensive caregiving involvement, characterized by high time commitment and frequent participation in multiple disease-management activities.

Following patient consent, clinical data on IBD diagnosis, disease duration, and current disease activity were retrieved from medical records. Disease activity referred to the patient’s clinical status at enrolment and was classified as remission, mild, moderate, or severe according to the criteria used in the participating IBD centres. This variable captured current disease activity at the time of data collection and did not measure cumulative disease severity, number of previous flares, or duration of flare episodes.

### Data collection procedures

During scheduled outpatient visits, research nurses trained in the IBD-SELF protocol identified eligible patient–caregiver dyads and approached potential caregiver participants. After oral and written information about the study had been provided, written informed consent was obtained from informal caregivers. Questionnaires could be completed either on paper during the clinic visit or electronically. The choice of administration mode was offered to reduce respondent burden, accommodate caregivers’ time constraints during outpatient visits, and facilitate participation across the nine centres. The paper and electronic versions contained the same items, response options, and instructions.

Completion time was reported as approximately 15 min. Paper questionnaires were returned to the clinic in sealed envelopes and subsequently entered into the study database by trained research staff. Electronic questionnaires were completed through a secure platform. In both modes, questionnaires were checked for completeness by a member of the research team before inclusion in the analytic dataset.

### Potential sources of bias

Potential selection bias was considered, as participation relied on caregivers’ willingness to take part during routine outpatient visits. Information bias may also arise from the use of self-reported questionnaires. To minimise these risks, standardised instruments with established validity were used, and data collection procedures were harmonised across centres. Information on non-respondents was limited to clinical variables of the corresponding patients. In particular, only data on disease activity were available for caregivers who declined participation. No relevant differences in disease activity distribution were observed between participants and non-participants. Data on caregivers’ relationship to the patient and care load among non-respondents were not available, as these variables were collected solely from participating caregivers.

### Data analysis

Analyses were performed using R, version 4.4.1 (R Core Team, [27]). The relationship between CC-SC-II Maintenance, Monitoring, and Management and caregivers’ HRQoL (SF-12 Physical Component Summary [PCS-12] and Mental Component Summary [MCS-12]) was investigated using Generalized Additive Models (GAMs) in the *mgcv* package [44].

GAMs are a flexible extension of the generalized linear model (GLM) framework that allow nonlinear relationships between predictors and outcomes to be estimated through smooth functions (smooth splines). Unlike linear models, which assume a constant rate of change across the predictor’s range, GAMs approximate complex, potentially curvilinear associations without pre-specifying their functional form. This flexibility is particularly useful in psychosocial and clinical research, where effects may not be linear—for instance, incremental increases in caregiving competence may improve mental health only up to a saturation point, beyond which gains are minimal or negative.

Formally, a GAM models the expected value of the outcome Y as:

  

$$\:g (E\left[ {\mathrm{Y}} \right]){\text{ }} = {\text{ }}\upbeta _{0} \, + \,{\mathrm{s}}_{{\mathrm{1}}} ({\mathrm{X}}_{{\mathrm{1}}} ) + {\mathrm{s}}_{{\mathrm{2}}} ({\mathrm{X}}_{{\mathrm{2}}} ) + \cdots + \varepsilon $$

where *g* is a link function (identity in our case), and $\left(\right)$ are smooth functions estimated by penalized regression splines. The degree of smoothness is selected automatically by restricted maximum likelihood (REML), balancing goodness-of-fit and model parsimony to prevent overfitting.

**Fig. 1:**
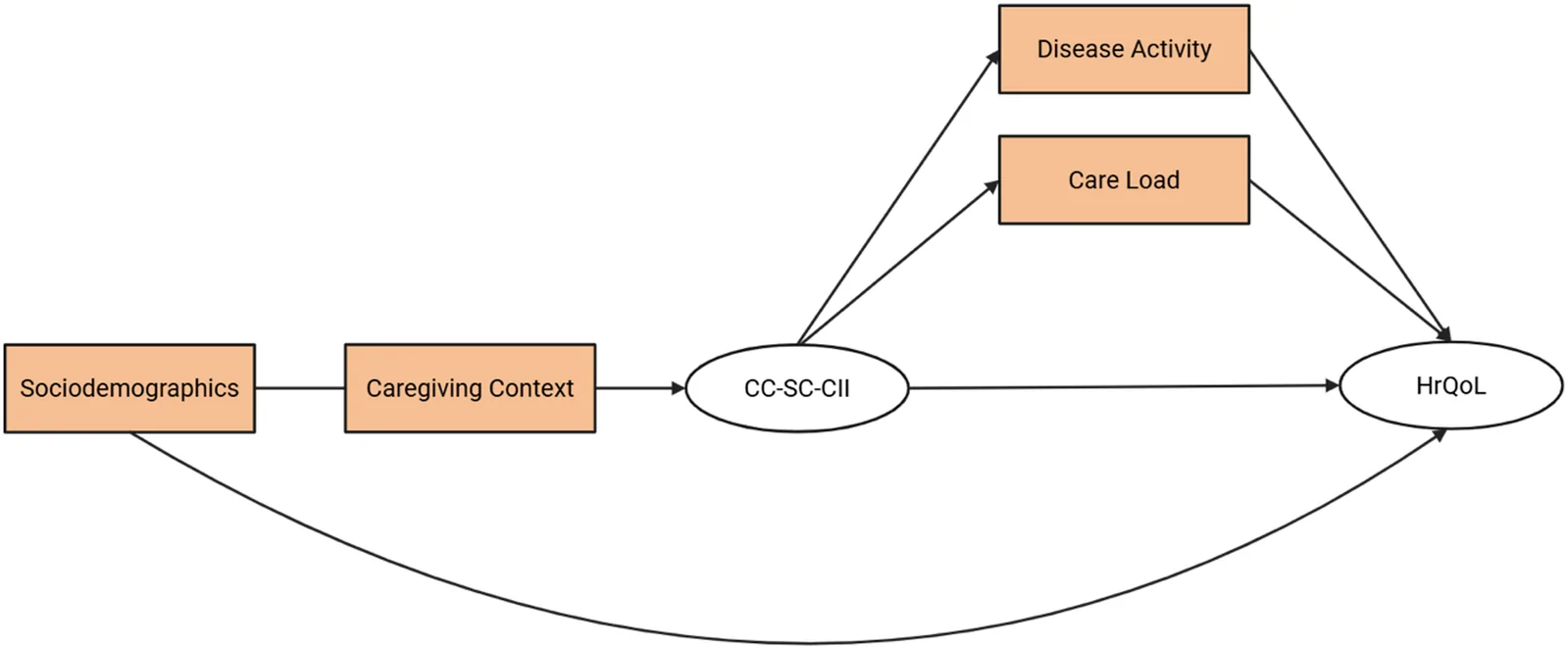
Directed acyclic graph (DAG) of caregiver contribution to patient self-care and health-related quality of life in IBD. *Note* Directed acyclic graph (DAG) depicting the hypothesised causal relationships among sociodemographic factors, caregiving context, caregiver contribution to self-care (CC-SC-CII), disease activity, care load, and caregivers’ health-related quality of life (HRQoL). Sociodemographic factors influence both caregiving context and HRQoL. The caregiving context shapes caregivers’ contributions to self-care, which is modelled as the central exposure and hypothesised to affect HRQoL directly and indirectly through downstream clinical and care-related variables. Disease activity and care load are represented as contextual variables measured at enrolment and potentially related to both the caregiving process and caregivers’ HRQoL. Given the cross-sectional design, the DAG was used to make covariate-selection assumptions explicit, not to establish temporal ordering or test mediation pathways. The DAG is grounded in dyadic self-care frameworks and the middle-range theory of self-care of chronic illness and was used to support causal reasoning and confounder selection

Potential confounding was examined using DAGs constructed with the dagitty package in R [34]. DAGs are graphical representations of hypothesized causal relationships between variables, where nodes represent variables and arrows represent directed causal effects. They are acyclic, meaning that no variable can causally influence itself through a feedback loop. DAG-based causal reasoning identifies which variables need to be statistically controlled (the minimal sufficient adjustment set) to obtain an unbiased estimate of the exposure–outcome relationship. In the present study, the DAG was constructed to represent theoretical and empirical knowledge about caregiving, where caregivers’ sociodemographic characteristics (e.g., age, sex, education, occupation, marital status) and contextual caregiving variables (e.g., relationship with the patient, time spent providing care, care load, and access to support services) may influence both caregivers’ competence and their HRQoL (Fig. 1).

DAG reasoning was used to make explicit the assumptions underlying covariate selection. The DAG was not intended to test mediation or to establish temporal pathways, which cannot be inferred from the present cross-sectional design. Instead, it was used to identify whether any measured variables should be included as covariates when estimating the primary cross-sectional association between each CC-SC-CII domain and caregivers’ HRQoL. In the specified DAG, caregiver sociodemographic characteristics and caregiving context were conceptualised as background factors that may shape both caregiver contribution and HRQoL. Disease activity and care load were considered contextual variables measured at enrolment and potentially related to both the caregiving process and caregiver HRQoL. However, because the temporal ordering among caregiver contribution, care load, disease activity, and HRQoL could not be established in this cross-sectional dataset, these variables were not interpreted as confirmed mediators. The *dagitty* analysis did not identify a non-empty minimally sufficient adjustment set for the primary association under the specified assumptions. In practical terms, this means that no measured covariate was selected as required to close an unblocked backdoor path between CC-SC-CII domains and HRQoL. Therefore, the primary GAMs were estimated without covariate adjustment and interpreted as unadjusted exploratory cross-sectional association models. This choice reflected the DAG-based covariate-selection assumptions and should not be interpreted as estimating a causal, direct, mediated, or individual-level predictive effect.

#### Study size

The sample size was determined by feasibility. No formal a priori power calculation was performed because the analysis was exploratory and no single prespecified effect size was available. However, the adequacy of the sample size was evaluated in relation to the complexity of the planned models. The DAG did not require a sample size calculation because it was not a fitted statistical model. The GAMs were parsimonious, with one smooth term per model and one HRQoL outcome at a time. The highest observed effective degrees of freedom was 4.34, indicating that the fitted smooths were modest relative to the sample size of 275. Thus, *n* = 275 was considered adequate for exploratory estimation of one-dimensional cross-sectional associations, but not for complex predictive, subgroup, or interaction modelling.

### Ethical considerations

The study protocol was reviewed and approved by the Territorial Ethics Committee Lazio 3 [Approval No. 0023486/23, dated August 2, 2023]. The study adhered to the ethical principles of the Declaration of Helsinki. Data were anonymised by assigning unique identifiers and stored on secure servers accessible only to authorised research staff.

## Results

### Characteristics of the sample

The study included 275 informal caregivers. The mean caregiver age was 51.6 years (SD = 9.5), and 160 caregivers were female (58.2%). Most caregivers were partners or spouses of the patient (*n* = 147, 53.5%), and 121 caregivers (44.0%) had been providing care for more than five years. Care load was most frequently classified as moderate (*n* = 136, 49.5%), followed by mild (*n* = 103, 37.5%) and severe (*n* = 36, 13.1%). More than half of the corresponding patients were in remission at enrolment (*n* = 149, 54.2%).

Mean CC-SC-CII scores were 55.33 (SD = 28.89) for Maintenance, 70.78 (SD = 33.33) for Monitoring, and 57.73 (SD = 24.44) for Management. Monitoring was the only domain with a mean score above the commonly used adequacy threshold of 70 on the 0–100 CC-SC-CII scale. Mean PCS-12 was 47.47 (SD = 6.04), and mean MCS-12 was 39.96 (SD = 5.89). Compared with the SF-12 norm-based reference mean PCS-12 was slightly lower, whereas MCS-12 was approximately one standard deviation lower.

**Table 1:** Sociodemographic characteristics of the sample

Variable	Values
Caregiver age (years) M (SD)	51.6 (9.5)
*Caregiver sex* n (%)	
Female	160 (58.2%)
Male	115 (41.8%)
*Caregiver care load* n (%)	
Mild	103 (37.5%)
Moderate	136 (49.5%)
Severe	36 (13.1%)
*Caregiver education level* n (%)	
Primary school	7 (2.5%)
Middle school	61 (22.2%)
High school	134 (48.7%)
Bachelor’s degree	73 (26.5%)
*Caregiver work status* n (%)	
Worker	184 (66.9%)
Non-worker (homemaker, retired, etc.)	91 (33.1%)
*Relationship to patient* n (%)	
Partner/spouse	147 (53.5%)
Parent/sibling	50 (18.2%)
Other family member	26 (9.5%)
Friend/other	29 (10.5%)
*Patient’s disease activity* n (%)	
Remission	149 (54.2%)
Mild	38 (13.8%)
Moderate	46 (16.7%)
Severe	42 (15.3%)
*Time as caregiver* n (%)	
< 1 year	64 (23.3%)
1–2.9 years	50 (18.2%)
3–4.9 years	40 (14.5%)
≥ 5 years	121 (44.0%)
*Caregiver contribution to patient self-care* M (SD)	
CC-SC-CII maintenance	55.33 (28.89)
CC-SC-CII monitoring	70.78 (33.33)
CC-SC-CII management	57.73 (24.44)
*Quality of life* M (SD)	
PCS-12	47.47 (6.04)
MCS-12	39.96 (5.89)

CC-SC-CII, caregiver contribution to patient self-care of chronic illness inventory; PCS-12, physical component summary; MCS-12, Mental Component Summary. Percentages may not be 100% due to rounding. Relationship-to-patient data were missing for 23 caregivers (8.4%).

### Association between HRQoL and CC-SC-II

#### Association between caregiver contribution to patient self-care and physical health (PCS-12)

GAMs showed no evidence of association (Table 2) between any CC-SC-CII dimension and PCS-12. The smooth terms for CC-SC-CII Maintenance, Monitoring, and Management were not supported (all *p* > 0.12), and model fit was poor (adjusted R² ranging from − 0.03 to 0.05; deviance explained 0.6–8.4%).

**Table 2:** Summary of GAM models estimating PCS-12

Predictor	edf	F	*p*	Adj. *R*²	Deviance explained (%)	25th 75th percentile		Δ(75 − 25)	95% CI lower	95% CI upper
CC-SC-CII maintenance	1.00	2.56	0.121	0.051	8.39	46.4	74.1	− 2.40	− 5.35	0.54
CC-SC-CII monitoring	1.00	0.18	0.676	− 0.029	0.64	51.2	100.0	− 0.69	− 3.89	2.51
CC-SC-CII management	1.26	0.56	0.651	− 0.008	3.56	50.0	75.0	− 0.57	− 3.43	2.28

EDF, effective degrees of freedom for the smooth term; F, approximate F statistic for the smooth function; *p*-values refer to the significance of the smooth term. Adjusted R² represents the proportion of variance in the outcome explained by the model, corrected for the number of predictors. Deviance Explained = percentage of variability in the dependent variable accounted for by the fitted GAM. All models were estimated using restricted maximum likelihood (REML)

CC-SC-CII, caregiver contribution to patient self-care inventory; EDF, effective degrees of freedom; GAM, generalized additive model; PCS-12, physical component summary

Primary GAMs were estimated without covariate adjustment. The DAG analysis did not identify a non-empty minimally sufficient adjustment set under the specified assumptions; therefore, no measured covariate was selected as required for the primary cross-sectional association. These models should not be interpreted as predictive or causal models.

#### Association between caregiver contribution to patient self-care and mental health (MCS-12)

The association between MCS-12 and CC-SC-CII Maintenance was non-linear (edf = 4.34) and significant in the global smooth test (F = 2.21, *p* = 0.018), with moderate model fit (adjusted R² = 0.27; deviance explained = 38.1%). No evidence of association was observed for CC-SC-CII Monitoring or Management (both *p* > 0.20).

**Fig. 2:**
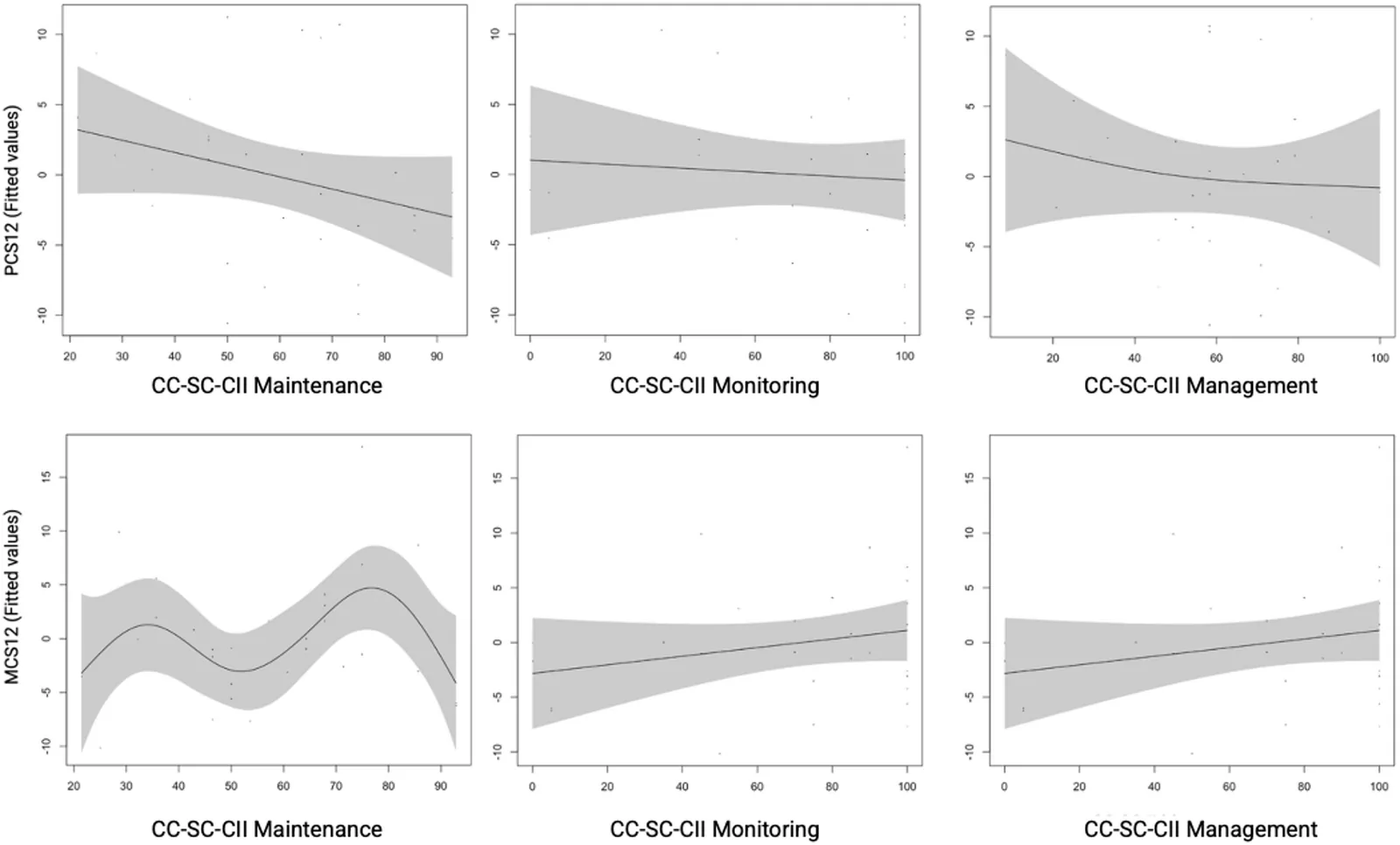
Smooth functions estimated from GAMs examining the associations between caregivers’ contribution dimensions (Maintenance, Monitoring, and Management) and HRQoL outcomes (PCS-12 and MCS-12). The top row displays models with PCS-12 (physical component summary) as the outcome variable, while the bottom row displays models with MCS-12 (Mental Component Summary) as the outcome variable. Solid lines represent the fitted smooth functions, and shaded areas indicate 95% confidence intervals. Data points denote partial residuals. A non-linear (spline-based smooth) significant trend is observable for the association between CC-SC-CII Maintenance and MCS-12, suggesting a possible curvilinear relationship between contribution to patient self-caremaintenance and caregivers’ mental health. CC-SC-CII = Caregiver contribution to patient self-care of chronic illness inventory. PCS = Physical component summary. MCS = Mental component summary.

Moving from the 25th to the 75th percentile of CC-SC-CII Maintenance (46.4 to 74.1) was associated with a + 6.6-point higher MCS-12 (95% CI 1.5, 11.7). Estimated MCS-12 values increased from 37.8 (95% CI 34.4, 41.2) at the 25th percentile to 44.4 (95% CI 40.8, 48.0) at the 75th percentile of CC-SC-CII Maintenance (Table 3). Corresponding contrasts for Monitoring and Management were not supported (Fig. 2).

**Table 3:** Summary of GAM models estimating MCS-12

Predictor	edf	F	*p*	Adj. *R*²	Deviance explained (%)	25th	75th	Δ(75–25)	95% CI lower	95% CI upper
CC-SC-CII Maintenance	4.34	2.21	0.018	0.272	38.10	46.4	74.1	6.58	1.50	11.70
CC-SC-CII Monitoring	1.00	1.51	0.229	0.017	5.12	51.2	100.0	1.91	−1.14	4.97
CC-SC-CII Management	1.73	0.22	0.740	0.007	6.66	50.0	75.0	−1.44	−4.68	1.80

EDF, effective degrees of freedom for the smooth term; F, approximate F statistic for the smooth function; *p*-values refer to the significance of the smooth term. Adjusted R² represents the proportion of variance in the outcome explained by the model, corrected for the number of predictors. Deviance explained = percentage of variability in the dependent variable accounted for by the fitted GAM.

CC-SC-CII, caregiver contribution to patient self-care inventory

Model visualization is depicted in Fig. 2.

#### Derivative-based analyses of the CC-SC-CII maintenance smooth

Using derivative-based analyses with simultaneous 95%CIs, no regions were detected in which the slope of the CC-SC-CII Maintenance smooth differed from zero (i.e., the simultaneous 95% CIs for the derivative included zero across the evaluated range). The derivative was negative for high CC-SC-CII Maintenance values (approximately 83.1–89.7), with pointwise 95% CIs entirely below zero (e.g., at Maintenance = 85.5, dŷ/dx = − 0.569, SE = 0.278, 95% CI [− 1.200, − 0.013]). This pattern suggests a potential decline in caregiver mental health at very high levels of CC-SC-CII Maintenance.

### Descriptive summary of cross-sectional findings

The GAMs did not show statistically supported associations between any CC-SC-CII domain and PCS-12. This finding should not be interpreted as evidence that physical HRQoL is unimportant or unrelated to the caregiving experience in IBD, but only as the absence of a statistically supported association between the CC-SC-CII domains and PCS-12 in the present models. For MCS-12, CC-SC-CII Maintenance was the only domain showing a statistically supported non-linear association (edf = 4.34, F = 2.21, *p* = 0.018). The model-estimated difference in MCS-12 between the 75th and 25th percentiles of CC-SC-CII Maintenance was 6.58 points (95% CI 1.50 to 11.70). No statistically supported associations were observed for CC-SC-CII Monitoring or Management.

## Discussion

This study provides an innovative perspective on the well-being of informal caregivers of adults with IBD, showing that their contribution to patient self-care is linked to mental, but not physical, HRQoL in a non-linear way. Specifically, only the maintenance dimension of caregiver contribution was associated with mental QoL (MCS-12), and this association followed an S-shaped pattern, whereas no dimension was associated with physical QoL (PCS-12). Framed within the Middle-Range Theory of Self-Care of Chronic Illness [28], these findings suggest that the impact of caregiving on mental health cannot be captured by simple “more is better” assumptions and may vary across levels of caregiving competence and involvement.

Visually, the smooth for contribution to self-care maintenance showed a wave-like (e.g., bimodal) pattern: mental HRQoL increased from low maintenance values to a first local maximum (approximately in the 30–40 range), then decreased toward a mid-range trough (around 50–60), followed by a more pronounced increase to a second peak (around 70–80), and finally a decline at the highest maintenance scores. Across the interquartile range, expected MCS-12 was higher at the 75th vs. the 25th percentile (74.1 vs. 46.4), corresponding to an estimated + 6.6-point difference, while uncertainty widened at the extremes.

The curvilinear association is compatible with stress-process and caregiving frameworks, in which caregiving can be simultaneously demanding and potentially protective, depending on intensity, appraisal, and available resources [31]. In IBD, caregiving demands can be persistent and fluctuate with symptoms, treatment routines, and uncertainty, which may shape caregivers’ emotional responses over time [32]. In this context, higher engagement in routine support may be associated with better psychological adjustment for some caregivers, while still posing a risk for others when demands become excessive [3, 35]. One interpretation of the observed pattern is that, for many caregivers, greater contribution to self-care maintenance behaviors may be linked with increased mastery and role integration, mechanisms that can support mental well-being. This aligns with work describing how caregivers can develop resilience and meaning-making alongside stress, particularly when they perceive competence and support [46]. Framing caregiver activity as “contribution to the patient’s self-care” (rather than burden alone) is also consistent with contemporary conceptualisations of self-care as a shared process in chronic illness [9]. At higher levels of involvement, caregivers may develop adaptive strategies that help maintain psychological balance despite persistent demands [46, 49].

A second relevant finding is that only the contribution to the self-care maintenance dimension showed evidence of association with mental HRQoL, while monitoring and management did not. A likely explanation is that maintenance reflects stable, day-to-day routines that shape caregivers’ ongoing experience, whereas monitoring and management are often episodic, flare-driven, and highly dependent on acute events; their emotional impact may therefore be transient or timing-dependent in a single time-point assessment. This distinction aligns with the Middle-Range Theory of Self-Care of Chronic Illness, in which maintenance represents sustained behaviours, whereas monitoring and management may occur intermittently as symptoms change [28]. In IBD, routine support behaviours are commonly embedded in daily life and family organisation, potentially making maintenance the dimension most consistently linked to caregivers’ psychological experience [4, 9, 16]. In addition, emerging evidence suggests that caregiver involvement and its consequences can vary substantially by context and role distribution within the dyad and the wider care network [19, 39].

From a clinical perspective, the unadjusted nature of the primary models should be interpreted. The observed association between CC-SC-CII Maintenance and MCS-12 does not imply that increasing caregiver contribution would necessarily improve caregivers’ mental HRQoL, nor does it provide an individual-level prediction for specific caregiver profiles. Rather, it suggests that the way caregivers are involved in routine maintenance activities may be relevant to their perceived mental health and may therefore deserve attention during clinical assessment. Importantly, the absence of statistically supported associations between the CC-SC-CII domains and PCS-12 should not be interpreted as evidence that physical HRQoL is unimportant or unrelated to the caregiving experience in IBD. Rather, the present analyses did not identify statistically supported cross-sectional associations within the current sample and modelling framework. For IBD services, the practical implication is therefore not to increase caregiver involvement indiscriminately, but to assess whether maintenance-related caregiving is experienced as manageable, meaningful, or burdensome, and to identify caregivers who may need tailored support. Our results fit with the evolving IBD caregiving literature, which is moving beyond an exclusive focus on burden toward a more nuanced view that includes adaptation, shared responsibility, and caregiver resources [19, 46]. Recognising that burden and positive adaptation can co-occur may help reconcile mixed evidence across studies and explain why caregiving is not uniformly detrimental [43]. Recent IBD studies likewise highlight the importance of caregiver support, coping, and relational dynamics for outcomes on both sides of the dyad [3, 35, 49]. Within this perspective, the present findings support a shift from asking whether caregivers are involved to understanding how, and with what psychological correlates.

These findings highlight a potential expanded role for the IBD nurse in implementing caregiver-inclusive models of care [45]. Positioned at the interface between patients, caregivers, and the multidisciplinary team, the IBD nurse is well-suited to assess not only whether caregivers are involved but also how they contribute to daily self-care and how this involvement affects their psychological well-being [10, 23]. Such a perspective supports the development of nurse-led pathways that tailor support to different patterns of caregiving engagement, moving beyond one-size-fits-all approaches to caregiver support.

### Strengths and Limitations

Methodologically, the study combines a theory-based measurement with flexible modeling to explore non-linearity without imposing arbitrary cut-offs. The use of causal diagrams to guide covariate decisions provides transparency about the assumed structure and the rationale for unadjusted models. Nevertheless, important limitations remain: the cross-sectional design precludes causal inference and does not establish directionality; self-report may introduce common-method bias; and unmeasured factors (e.g., social support, coping style, relationship quality) may influence both contribution and HRQoL. At the same time, the non-linear finding should be interpreted with appropriate caution. While the global smooth test supported nonlinearity, conservative derivative inference did not identify clear ranges in which the slope differed from zero across the continuum; pointwise derivatives suggested a possible decline at very high maintenance scores. Therefore, any “downturn” at the extreme upper end should be considered hypothesis-generating rather than confirmatory. Clinically, this remains plausible: very high and sustained involvement can translate into role overload, emotional exhaustion, and reduced personal resources, especially when support is limited [2, 29]. In other chronic illness contexts, such strain has been linked with caregiver health risks and reduced well-being, reinforcing the need to monitor the most intensely involved caregivers [1, 29]. Another measurement-related consideration concerns the instruments used. The CC-SC-CII is a relatively recent instrument designed to assess caregiver contribution to patient self-care, whereas the SF-12 is a generic HRQoL instrument whose Italian validation was published in 2001. Although the SF-12 remains widely used and enables comparison with broader HRQoL literature, its generic structure may not fully capture caregiving-specific aspects of quality of life or contemporary dimensions of caregiver well-being. The relatively low MCS-12 scores observed in the present sample may reflect the psychological demands associated with long-term caregiving in chronic fluctuating conditions such as IBD. Interestingly, the original Italian validation study of the SF-12 reported that the Mental Component Summary was less sensitive to age-related variation than the Physical Component Summary [13]. This suggests that the reduced MCS-12 scores observed in the current study are unlikely to be explained solely by caregiver age and may instead reflect broader psychological and caregiving-related factors. Therefore, PCS-12 and MCS-12 should be interpreted as standardised caregiver-reported perceptions of physical and mental health status, rather than objective measures of physical or psychological functioning. This is particularly relevant in a cross-sectional study, where perceived HRQoL may reflect both stable caregiver characteristics and temporary contextual conditions at the time of assessment.

Although the Italian validation of the SF-12 was conducted more than two decades ago, the instrument continues to be widely used in HRQoL research and provides a standardised framework for comparing perceived physical and mental health across populations. Nevertheless, possible temporal changes in population norms and caregiving experiences should be considered when interpreting the present findings.

### Implications for practice and research

These findings have clinical implications. Services may benefit from routinely assessing not only whether caregivers are involved, but also how they contribute to daily maintenance work, and from offering support that helps distribute tasks, build skills, and protect caregivers’ psychological well-being. In addition to psychoeducation and practical training, interventions combining problem-solving with stress-management components may be particularly relevant when demands are sustained [48]. For patients, caregiver-inclusive approaches may improve continuity of self-care routines, treatment adherence, symptom monitoring, and timely help-seeking behaviours during disease exacerbations. For families, recognising caregiving as a dynamic and shared process may facilitate communication, reduce role ambiguity, and support more adaptive coping strategies within the household. For healthcare services, integrating caregiver assessment into routine IBD care may help identify caregivers at risk of psychological distress, allowing earlier supportive interventions and potentially reducing avoidable service utilisation linked to caregiving strain or inadequate disease management support. From a broader integrated-care perspective, collaboration between IBD teams, primary care providers, mental health professionals, and social services may support caregivers facing sustained emotional, organisational, or socioeconomic burden. Examples may include referral pathways to social workers, caregiver financial-support resources, community-based assistance programmes, caregiver support groups, and integrated psychosocial services tailored to chronic gastrointestinal conditions. From a health-system perspective, caregiver support can have downstream benefits for families and services and should be considered in planning sustainable models of chronic care [37].

The present findings also align with evidence from other chronic illnesses, including heart failure, oncology, and neurodegenerative diseases, where caregiver involvement has been associated with both adaptive psychological processes and increased emotional burden depending on caregiving intensity and contextual resources [4, 8, 16]. Compared with these conditions, IBD caregiving may present additional challenges related to disease unpredictability, fluctuating symptom burden, dietary management, and uncertainty surrounding relapses, all of which may shape caregiver contribution and psychological adaptation differently. This supports the importance of interpreting caregiving in IBD not exclusively through burden-oriented frameworks, but also through relational and self-care-oriented perspectives.

Future longitudinal research is needed to clarify temporal relationships between caregiver contribution and mental HRQoL, to identify potential moderators, and to test whether caregiver-inclusive interventions that optimise daily maintenance routines can improve caregivers’ mental HRQoL and mitigate burnout over time. Prospective designs would also help determine whether the potential decline at very high maintenance contribution reflects a meaningful risk signal or a cross-sectional artefact, and whether psychological effects emerge gradually [6, 31].

## Conclusion

In summary, this study suggests that caregiver contribution to patient self-care maintenance in IBD is related to caregivers’ mental, but not physical, HRQoL in a complex, non-linear fashion, with heterogeneous psychological correlates across different levels of caregiving involvement. These findings provide a conceptual and empirical rationale for developing caregiver-inclusive interventions tailored to different levels of caregiving involvement in IBD and underscore the need for rigorous longitudinal and interventional research to determine whether appropriately targeted caregiver support strategies can sustainably protect caregivers’ mental health.

## Supplementary Information

Below is the link to the electronic supplementary material.

Supplementary Material 1

## Data Availability

The datasets generated during and/or analysed during the current study are available from the corresponding author on reasonable request.

## References

[1] Adelman, R. D., Tmanova, L. L., Delgado, D., Dion, S., & Lachs, M. S. (2014). Caregiver burden: A clinical review. *Journal Of The American Medical Association*, *311*(10), 1052–1060. 10.1001/jama.2014.304 24618967

[2] Applebaum, A. J., & Breitbart, W. (2013). Care for the cancer caregiver: A systematic review. *Palliative & Supportive Care*, *11*(3), 231–252. 10.1017/S1478951512000594 23046977 PMC4973511

[3] Bozzetti, M., Marcomini, I., Lo Cascio, A., Magurano, M. R., Ribaudi, E., Petralito, M., Milani, I., Amato, S., Orgiana, N., Parello, S., Puca, P., Scaldaferri, F., Mazza, M., Marano, G., & Napolitano, D. (2025). Exploring the mediating role of self-efficacy in the relationship between caregiver contribution and resilience in inflammatory bowel disease. *Behavioral Sciences*, *15*(10), 1381. 10.3390/bs15101381 41153171 PMC12561742

[4] Caggianelli, G., Alivernini, F., Chirico, A., Iovino, P., Lucidi, F., Uchmanowicz, I., Rasero, L., Alvaro, R., & Vellone, E. (2024). The relationship between caregiver contribution to self-care and patient quality of life in heart failure: A longitudinal mediation analysis. *PloS One*, *19*(3), e0300101. 10.1371/journal.pone.0300101 38470867 PMC10931462

[5] Caron, B., Honap, S., & Peyrin-Biroulet, L. (2024). Epidemiology of inflammatory bowel disease across the ages in the era of advanced therapies. *Journal of Crohn’s & Colitis*, *18*(Supplement_2), ii3–ii15. 10.1093/ecco-jcc/jjae082 PMC11522978 39475082

[6] Chhibba, T., Gros, B., King, J. A., Windsor, J. W., Gorospe, J., Leibovitzh, H., Xue, M., Turpin, W., Croitoru, K., Ananthakrishnan, A. N., Gearry, R. B., & Kaplan, G. G. (2025). Environmental risk factors of inflammatory bowel disease: Toward a strategy of preventative health. *Journal of Crohn’s & Colitis*, *19*(4), jjaf042. 10.1093/ecco-jcc/jjaf042 PMC12010164 40065502

[7] Coward, S., Clement, F., Benchimol, E. I., Bernstein, C. N., Avina-Zubieta, J. A., Bitton, A., Carroll, M. W., Hazlewood, G., Jacobson, K., Jelinski, S., Deardon, R., Jones, J. L., Kuenzig, M. E., Leddin, D., McBrien, K. A., Murthy, S. K., Nguyen, G. C., Otley, A. R., Panaccione, R., & Kaplan, G. G. (2019). Past and future burden of inflammatory bowel diseases based on modeling of population-based data. *Gastroenterology*, *156*(5), 1345–1353e4. 10.1053/j.gastro.2019.01.002 30639677

[8] Cui, P., Yang, M., Hu, H., Cheng, C., Chen, X., Shi, J., Li, S., Chen, C., & Zhang, H. (2024). The impact of caregiver burden on quality of life in family caregivers of patients with advanced cancer: A moderated mediation analysis of the role of psychological distress and family resilience. *Bmc Public Health*, *24*(1), 817. 10.1186/s12889-024-18321-3 38491454 PMC10941369

[9] De Maria, M., Ausili, D., Lorini, S., Vellone, E., Riegel, B., & Matarese, M. (2022). Patient Self-Care and Caregiver Contribution to Patient Self-Care of Chronic Conditions: What Is Dyadic and What It Is Not. *Value in Health: The Journal of the International Society for Pharmacoeconomics and Outcomes Research*, *25*(7), 1165–1173. 10.1016/j.jval.2022.01.007 35337754

[10] Fiorino, G., Caprioli, F. A., Onali, S., Macaluso, F. S., Bezzio, C., Armelao, F., Armuzzi, A., Baldoni, M., Bodini, G., Castiglione, F., Daperno, M., Festa, S., Furfaro, F., Gionchetti, P., Leone, S., Luglio, G., Milla, M., Mocci, G., Napolitano, D., & Fantini, M. C. (2025). Adaptation of the European Crohn’s Colitis Organisation quality of care standards to Italy: The Italian Group for the study of inflammatory bowel disease consensus. *Digestive and Liver Disease: Official Journal of the Italian Society of Gastroenterology and the Italian Association for the Study of the Liver*. 10.1016/j.dld.2025.03.010. S1590-8658(25)00288-9. 40189424

[11] Iizawa, M., Hirose, L., Nunotani, M., Nakashoji, M., Tairaka, A., & Fernandez, J. L. (2023). A systematic review of self-management interventions for patients with inflammatory bowel disease. *Inflammatory Intestinal Diseases*, *8*(1), 1–12. 10.1159/000530021 37404383 PMC10315013

[12] Iovino, P., Lyons, K. S., De Maria, M., Vellone, E., Ausili, D., Lee, C. S., Riegel, B., & Matarese, M. (2021). Patient and caregiver contributions to self-care in multiple chronic conditions: A multilevel modelling analysis. *International Journal of Nursing Studies*, *116*, 103574. 10.1016/j.ijnurstu.2020.103574 32276720

[13] Kodraliu, G., Mosconi, P., Groth, N., Carmosino, G., Perilli, A., Gianicolo, E. A., Rossi, C., & Apolone, G. (2001). Subjective health status assessment: Evaluation of the Italian version of the SF-12 Health Survey. Results from the MiOS Project. *Journal of Epidemiology and Biostatistics*, *6*(3), 305–316. 10.1080/135952201317080715 11437095

[14] Kodraliu, P., Mosconi, N., & Groth, G., G (2001). Subjective health status assessment: Evaluation of the Italian version of the SF-12 Health Survey. Results from the MiOS project. *Journal of Epidemiology and Biostatistics*, *6*(3), 305–316. 10.1080/135952201317080715 11437095

[15] Litzelman, K. (2019). Caregiver well-being and the quality of cancer care. *Seminars in Oncology Nursing*, *35*(4), 348–353. 10.1016/j.soncn.2019.06.006 31229346 PMC6728914

[16] Locatelli, G., Iovino, P., Jurgens, C. Y., Alvaro, R., Uchmanowicz, I., Rasero, L., Riegel, B., & Vellone, E. (2024). The influence of caregiver contribution to self-care on symptom burden in patients with heart failure and the mediating role of patient self-care: A longitudinal mediation analysis. *The Journal of Cardiovascular Nursing*, *39*(3), 255–265. 10.1097/JCN.0000000000001024 37550831

[17] Locatelli, G., Rebora, P., Occhino, G., Ausili, D., Riegel, B., Cammarano, A., Uchmanowicz, I., Alvaro, R., Vellone, E., & Zeffiro, V. (2023). The impact of an intervention to improve caregiver contribution to heart failure self-care on caregiver anxiety, depression, quality of life, and sleep. *The Journal of Cardiovascular Nursing*, *38*(4), 361–369. 10.1097/JCN.0000000000000998 37204336 PMC10259208

[18] Manalel, J. A., Sumrall, S., Davidson, H., Grewal, M., Granovetter, M. A., & Koehly, L. M. (2024). Stress, coping, and positive aspects of caregiving among caregivers of children with rare disease. *Psychology & Health*, *39*(2), 216–232. 10.1080/08870446.2022.2057494 35620936 PMC9701241

[19] Mendiolaza, M., Ogundipe, T., Arroyave-Villada, J., Adeonigbagbe, O., Gorbenko, K., & Keefer, L. (2024). Investigating resilience in patients with IBD: Preliminary insights for understanding disease-specific resilience skills. *Frontiers in Psychology*, *15*, 1486401. 10.3389/fpsyg.2024.1486401 39606191 PMC11599970

[20] Mohsenizadeh, S. M., Manzari, Z. S., Vosoghinia, H., & Ebrahimipour, H. (2020). Family caregivers’ burden in inflammatory bowel diseases: An integrative review. *Journal of Education and Health Promotion*, *9*, 289. 10.4103/jehp.jehp_233_20 33282994 PMC7709749

[21] Napolitano, D., Biagioli, V., Bartoli, D., Cilluffo, S., Martella, P., Monaci, A., Vellone, E., & Cocchieri, A. (2025). Validity and Reliability of the Self-Care of Chronic Illness Inventory in Patients Living With Inflammatory Bowel Disease. *Journal of Clinical Nursing*. 10.1111/jocn.17712 40033448

[22] Napolitano, D., Cilluffo, S., Amatucci, V., Bartoli, D., Biagioli, V., Martella, P., Monaci, A., Cocchieri, A., & Vellone, E. (2025). Self-care in patients with inflammatory bowel disease: A descriptive cross-sectional multicenter study. *Crohn’s & Colitis*, *360*, otaf061. 10.1093/crocol/otaf061 PMC12602154 41230340

[23] Napolitano, D., Schiavoni, E., & Scaldaferri, F. (2022). Nurse practitioners in inflammatory bowel disease: The emerging role of the IBD care manager. *Journal of Gastrointestinal and Liver Diseases: JGLD*, *31*(4), 4627. 10.15403/jgld-4627 36535047

[24] Napolitano, D., Vellone, E., Iovino, P., Scaldaferri, F., & Cocchieri, A. (2024). Self-care in patients affected by inflammatory bowel disease and caregiver contribution to self-care (IBD-SELF): A protocol for a longitudinal observational study. *BMJ Open Gastroenterology*, *11*(1), e001510. 10.1136/bmjgast-2024-001510 39209770 PMC13059948

[25] Niu, S., Ding, S., Wu, S., Ma, J., & Shi, Y. (2023). Correlations between caregiver competence, burden and health-related quality of life among Chinese family caregivers of elderly adults with disabilities: A cross-sectional study using structural equations analysis. *British Medical Journal Open*, *13*(2), e067296. 10.1136/bmjopen-2022-067296 PMC9944642 36806142

[26] Parekh, N. K., Shah, S., McMaster, K., Speziale, A., Yun, L., Nguyen, D. L., Melmed, G., & Kane, S. (2017). Effects of caregiver burden on quality of life and coping strategies utilized by caregivers of adult patients with inflammatory bowel disease. *Annals of Gastroenterology*, *30*(1), 89–95. 10.20524/aog.2016.0084 28042243 PMC5198253

[27] R Core Team (2023). *R: A language and environment for statistical computing* [Software]. R Foundation for Statistical Computing. https://www.R-project.org/.

[28] Riegel, B., Jaarsma, T., & Strömberg, A. (2012). A middle-range theory of self-care of chronic illness. *ANS Advances in Nursing Science*, *35*(3), 194–204. 10.1097/ANS.0b013e318261b1ba 22739426

[29] Roth, D. L., Fredman, L., & Haley, W. E. (2015). Informal caregiving and its impact on health: A reappraisal from population-based studies. *The Gerontologist*, *55*(2), 309–319. 10.1093/geront/gnu177 26035608 PMC6584119

[30] Sarter, H., Crétin, T., Savoye, G., Fumery, M., Leroyer, A., Dauchet, L., Paupard, T., Coevoet, H., Wils, P., Richard, N., Turck, D., Ley, D., Gower-Rousseau, C., & EPIMAD study Group. (2024). Incidence, prevalence and clinical presentation of inflammatory bowel diseases in Northern France: A 30-year population-based study. *The Lancet Regional Health Europe*, *47*, 101097. 10.1016/j.lanepe.2024.101097 39478988 PMC11522416

[31] Schulz, R., & Sherwood, P. R. (2008). Physical and mental health effects of family caregiving. *The American Journal of Nursing*, *108*(9 Suppl), 23–27. 10.1097/01.NAJ.0000336406.45248.4c. 18797217 PMC2791523

[32] Taft, T. H., & Aswani-Omprakash, T. (2024). Caregiver burden of IBD patients in Asian Emerging Nations is significant and necessitates attention and resource allocation. *Indian Journal of Gastroenterology: Official Journal of the Indian Society of Gastroenterology*, *43*(6), 1086–1089. 10.1007/s12664-024-01653-8 39088167

[33] Tennant, P. W. G., Murray, E. J., Arnold, K. F., Berrie, L., Fox, M. P., Gadd, S. C., Harrison, W. J., Keeble, C., Ranker, L. R., Textor, J., Tomova, G. D., Gilthorpe, M. S., & Ellison, G. T. H. (2021). Use of directed acyclic graphs (DAGs) to identify confounders in applied health research: Review and recommendations. *International Journal of Epidemiology*, *50*(2), 620–632. 10.1093/ije/dyaa213 33330936 PMC8128477

[34] Textor, J., Hardt, J., & Knüppel, S. (2011). DAGitty: A graphical tool for analyzing causal diagrams. *Epidemiology (Cambridge Mass)*, *22*(5), 745. 10.1097/EDE.0b013e318225c2be 21811114

[35] Thapwong, P., Norton, C., Rowland, E., & Czuber-Dochan, W. (2025). N02 Supporting psychosocial and emotional well-being and building resilience in family members of people with IBD: RCT feasibility and acceptability study. *Journal of Crohn’s and Colitis*, *19*(Supplement_1), i2445. 10.1093/ecco-jcc/jjae190.1534

[36] Thompson, T., Ketcher, D., Gray, T. F., & Kent, E. E. (2021). The dyadic cancer outcomes framework: A general framework of the effects of cancer on patients and informal caregivers. *Social Science & Medicine (1982)*, *287*, 114357. 10.1016/j.socscimed.2021.114357 34500320 PMC8936416

[37] Van Houtven, C. H., Voils, C. I., & Weinberger, M. (2011). An organizing framework for informal caregiver interventions: Detailing caregiving activities and caregiver and care recipient outcomes to optimize evaluation efforts. *BMC Geriatrics*, *11*(1), 77. 10.1186/1471-2318-11-77 22107600 PMC3258201

[38] van Roij, J., Brom, L., Sommeijer, D., van de Poll-Franse, L., Raijmakers, N., & eQuiPe study group. (2021). Self-care, resilience, and caregiver burden in relatives of patients with advanced cancer: Results from the eQuiPe study. *Supportive Care in Cancer: Official Journal of the Multinational Association of Supportive Care in Cancer*, *29*(12), 7975–7984. 10.1007/s00520-021-06365-9 34215933 PMC8549961

[39] Vellone, E., Barbaranelli, C., Pucciarelli, G., Zeffiro, V., Alvaro, R., & Riegel, B. (2020). Validity and reliability of the caregiver contribution to self-care of heart failure index version 2. *The Journal of Cardiovascular Nursing*, *35*(3), 280–290. 10.1097/JCN.0000000000000655 32084084

[40] Vellone, E., Riegel, B., & Alvaro, R. (2019). A situation-specific theory of caregiver contributions to heart failure self-care. *The Journal of Cardiovascular Nursing*, *34*(2), 166–173. 10.1097/JCN.0000000000000549 30363017

[41] von Elm, E., Altman, D. G., Egger, M., Pocock, S. J., Gøtzsche, P. C., Vandenbroucke, J. P., & STROBE Initiative. (2007). Strengthening the reporting of observational studies in epidemiology (STROBE) statement: Guidelines for reporting observational studies. *BMJ (Clinical Research Ed)*, *335*(7624), 806–808. 10.1136/bmj.39335.541782.AD PMC2034723 17947786

[42] Ware, J., Kosinski, M., & Keller, S. D. (1996). A 12-item short-form health survey: Construction of scales and preliminary tests of reliability and validity. *Medical Care*, *34*(3), 220–233. 10.1097/00005650-199603000-00003 8628042

[43] Wennberg, A. M., Anderson, L. R., Cagnin, A., Chen-Edinboro, L. P., & Pini, L. (2023). How both positive and burdensome caregiver experiences are associated with care recipient cognitive performance: Evidence from the National Health and Aging Trends Study and National Study of Caregiving. *Frontiers in Public Health*, *11*, 1130099. 10.3389/fpubh.2023.1130099 36860389 PMC9969137

[44] Wood, S. N. (2011). Fast stable restricted maximum likelihood and marginal likelihood estimation of semiparametric generalized linear models. *Journal of the Royal Statistical Society Series B: Statistical Methodology*, *73*(1), 3–36. 10.1111/j.1467-9868.2010.00749.x

[45] Yu, N., Wu, K., Samyue, T., Fry, S., Stanley, A., Ross, A., Malcolm, R., Connell, W., Wright, E., Ding, N. S., Niewiadomski, O., Lust, M., Schulberg, J., Flanagan, E., Kamm, M. A., & Basnayake, C. (2024). Outcomes of a comprehensive specialist inflammatory bowel disease nursing service. *Inflammatory Bowel Diseases*, *30*(6), 960–969. 10.1093/ibd/izad145 37643766

[46] Yuan, Y., Wang, H., Song, X., Tan, W., Liu, Y., Liu, H., Hu, C., & Guo, H. (2025). Exploring the multidimensional impact of caregiver burden in patients with inflammatory bowel disease. *Frontiers in Public Health*. 10.3389/fpubh.2025.1528778. 40510583 PMC12158650

[47] Zand, A., Kim, B. J., van Deen, W. K., Stokes, Z., Platt, A., O’Hara, S., Khong, H., & Hommes, D. W. (2020). The effects of inflammatory bowel disease on caregivers: Significant burden and loss of productivity. *BMC Health Services Research*, *20*(1), 556. 10.1186/s12913-020-05425-w 32552803 PMC7302133

[48] Zhai, S., Chu, F., Tan, M., Chi, N. C., Ward, T., & Yuwen, W. (2023). Digital health interventions to support family caregivers: An updated systematic review. *Digital Health*, *9*, 20552076231171967. 10.1177/20552076231171967 37223775 PMC10201006

[49] Zhou, M., Wang, M., Luo, D., Sun, C., Bian, Q., Xu, J., & Lin, Z. (2024). The mediating role of resilience between caregiver burden and hope among patients with inflammatory bowel disease. *Nursing Open*, *11*(8), e70001. 10.1002/nop2.70001 39189092 PMC11347936

