# Supplementary material for: Caregiver contribution to patient self‑care and quality of life among informal carers of adult patients with inflammatory bowel disease: a cross‑sectional study
Source: Qual Life Res. 2026 Jun 15;35(8):197. doi: 10.1007/s11136-026-04305-w (PMC13269151; doi:10.1007/s11136-026-04305-w)
Supplement: Supplementary file 1 — Supplementary Material 1 [file 11136_2026_4305_MOESM1_ESM.docx]

STROBE Statement—checklist of items that should be included in reports of observational studies

|  | Item No. | Recommendation | Page  No. | Relevant text from manuscript |
| --- | --- | --- | --- | --- |
| **Title and abstract** | 1 | (*a*) Indicate the study’s design with a commonly used term in the title or the abstract | 1-2 | The study is explicitly described as a “multicentre cross-sectional study” in the abstract and methods. |
|  |  | (*b*) Provide in the abstract an informative and balanced summary of what was done and what was found | 3-4 | Structured abstract including Purpose, Methods, Results, and Conclusion. |
| Introduction | | | |  |
| Background/rationale | 2 | Explain the scientific background and rationale for the investigation being reported | 4-8 | The burden of caregiving in IBD and the gap regarding caregiver contribution to self-care and quality of life are clearly described. |
| Objectives | 3 | State specific objectives, including any prespecified hypotheses | 4-8 | The objective to evaluate the association between caregiver contribution to self-care and HRQoL is explicitly stated. |
| Methods | | | |  |
| Study design | 4 | Present key elements of study design early in the paper | 8-12 | Multicentre cross-sectional design specified, with study period (April–June 2024). |
| Setting | 5 | Describe the setting, locations, and relevant dates, including periods of recruitment, exposure, follow-up, and data collection | 8-12 | Nine Italian IBD outpatient centres are described, including recruitment period.  Participants |
| Participants | 6 | (*a*) *Cross-sectional study*—Give the eligibility criteria, and the sources and methods of selection of participants | 8-12 | Inclusion and exclusion criteria for informal caregivers are clearly described, along with recruitment procedures via outpatient clinics. |
| Variables | 7 | Clearly define all outcomes, exposures, predictors, potential confounders, and effect modifiers. Give diagnostic criteria, if applicable | 8-12 | Exposure variables (CC-SC-CII Maintenance, Monitoring, Management) and outcomes (SF-12 PCS and MCS) are clearly defined. |
| Data sources/ measurement | 8* | For each variable of interest, give sources of data and details of methods of assessment (measurement). Describe comparability of assessment methods if there is more than one group | 8-12 | Use of validated instruments (CC-SC-CII, SF-12) and data collection procedures |
| Bias | 9 | Describe any efforts to address potential sources of bias | 8-12 | Potential selection bias and information bias due to self-report are acknowledged and discussed. |
| Study size | 10 | Explain how the study size was arrived at | 8-12 | Sample size is based on feasibility and availability of participants during the study period; exploratory nature of the analysis is stated. |

Continued on next page

| Quantitative variables | 11 | Explain how quantitative variables were handled in the analyses. If applicable, describe which groupings were chosen and why | 13-18 | CC-SC-CII scores transformed to a 0–100 scale; SF-12 standardised scores described.  Statistical methods |
| --- | --- | --- | --- | --- |
| Statistical methods | 12 | (*a*) Describe all statistical methods, including those used to control for confounding | 13-18 | Generalized Additive Models described in detail  – Confounding assessed using Directed Acyclic Graphs  – No covariate adjustment justified by causal assumptions  – No missing data observed, therefore no imputation required |
|  |  | (*b*) Describe any methods used to examine subgroups and interactions | NA |  |
|  |  | (*c*) Explain how missing data were addressed | 13-18 | No Missing Data |
|  |  | *Cross-sectional study*—If applicable, describe analytical methods taking account of sampling strategy | NA |  |
|  |  | (*e*) Describe any sensitivity analyses | NA |  |
| Results | | | | |
| Participants | 13* | (a) Report numbers of individuals at each stage of study—eg numbers potentially eligible, examined for eligibility, confirmed eligible, included in the study, completing follow-up, and analysed | 13-18 | 275 caregivers participated (60.8%); recruitment flow described in text. |
|  |  | (b) Give reasons for non-participation at each stage | NA |  |
|  |  | (c) Consider use of a flow diagram | NA |  |
| Descriptive data | 14* | (a) Give characteristics of study participants (eg demographic, clinical, social) and information on exposures and potential confounders | 13-18 | Sociodemographic and clinical characteristics of caregivers and patients reported.  Outcome data |
| Outcome data | 15* | *Cohort study*—Report numbers of outcome events or summary measures over time | *NA* |  |
|  |  | *Case-control study—*Report numbers in each exposure category, or summary measures of exposure | *NA* |  |
|  |  | *Cross-sectional study—*Report numbers of outcome events or summary measures | 13-18 | PCS-12 and MCS-12 scores reported as means and standard deviations. |
| Main results | 16 | (*a*) Give unadjusted estimates and, if applicable, confounder-adjusted estimates and their precision (eg, 95% confidence interval). Make clear which confounders were adjusted for and why they were included | 13-18 | GAM estimates including EDF, F statistics, p-values, adjusted R², and deviance explained. |
|  |  | (*b*) Report category boundaries when continuous variables were categorized | NA |  |
|  |  | (*c*) If relevant, consider translating estimates of relative risk into absolute risk for a meaningful time period | NA |  |

Continued on next page

| Other analyses | 17 | Report other analyses done—eg analyses of subgroups and interactions, and sensitivity analyses | 13-18 | Causal assumptions evaluated using Directed Acyclic Graphs. |
| --- | --- | --- | --- | --- |
| Discussion | | | | |
| Key results | 18 | Summarise key results with reference to study objectives | 19-22 | Main findings summarised in relation to study objectives. |
| Limitations | 19 | Discuss limitations of the study, taking into account sources of potential bias or imprecision. Discuss both direction and magnitude of any potential bias | 19-22 | Cross-sectional design, self-reported measures, and sample size limitations discussed. |
| Interpretation | 20 | Give a cautious overall interpretation of results considering objectives, limitations, multiplicity of analyses, results from similar studies, and other relevant evidence | 19-22 | Findings interpreted in light of the Middle-Range Theory of Self-Care and caregiving literature. |
| Generalisability | 21 | Discuss the generalisability (external validity) of the study results | 19-22 | Clinical implications and potential transferability to other IBD settings discussed. |
| Other information | |  | | |
| Funding | 22 | Give the source of funding and the role of the funders for the present study and, if applicable, for the original study on which the present article is based | 19-22 | No funding declared. |

*Give information separately for cases and controls in case-control studies and, if applicable, for exposed and unexposed groups in cohort and cross-sectional studies.

**Note:** An Explanation and Elaboration article discusses each checklist item and gives methodological background and published examples of transparent reporting. The STROBE checklist is best used in conjunction with this article (freely available on the Web sites of PLoS Medicine at http://www.plosmedicine.org/, Annals of Internal Medicine at http://www.annals.org/, and Epidemiology at http://www.epidem.com/). Information on the STROBE Initiative is available at www.strobe-statement.org.
